# Editorial: Alternative Splicing in Health and Disease

**DOI:** 10.3389/fmolb.2022.878668

**Published:** 2022-04-19

**Authors:** Abdullah Kahraman, Marija Buljan, Kristoffer Vitting-Seerup

**Affiliations:** ^1^ University Hospital Zürich, Zurich, Switzerland; ^2^ Swiss Insitute of Bioinformatics, Lausanne, Switzerland; ^3^ Swiss Federal Laboratories for Materials Science and Technology, St. Gallen, Switzerland; ^4^ Section for Bioinformatics, Health Technology, The Technical University of Denmark (DTU), Lyngby, Denmark

**Keywords:** alternative splice, isoforms, review, diagnostics, annotation

Alternative splicing is an essential, ubiquitous molecular mechanism highly conserved throughout all multicellular eukaryotes. Along with alternative transcription start- and termination-sites, alternative splicing enables a single gene to produce multiple protein isoforms with distinct biological functions, thereby contributing significantly to the functional complexity of humans (Barbosa-Morais et al., 2012). Correct alternative splicing is essential to health and homeostasis, and when disrupted often leads to disease development (Bonnal et al., 2020). Although alternative splicing is increasingly studied, the landscape of its diverse functional roles and disease-associated dysregulations are still vastly under-investigated (Vitting-Seerup and Sandelin, 2017). This Research Topic adds to the ever-increasing body of evidence linking alternative splicing to a wide range of cellular observations and diseases. Zhou et al. provide a comprehensive review of how alternative splicing is involved in various pediatric liver diseases ranging from viral infections to tumors, highlighting specific genes and splicing mutations that contribute to the disease etiology. Elaborating on the cancer topic, Karakulak et al. review the molecular mechanisms that drive alternative splicing diversity in both healthy and tumor cells along with bioinformatic tools to assist the analysis of these changes. Karakulak and colleagues provide a long-overdue comparison of three recent studies that all performed pan-cancer analyses of isoform switches with predicted functional consequences (Climente-González et al., 2017; Vitting-Seerup and Sandelin, 2017; Kahraman et al., 2020). Although all three studies find isoform switches to be widespread and biologically relevant, their overlap is surprisingly low, indicating the importance of methodological choices for cataloging the isoforms and analyzing their function.

The origins of alternatively spliced exons are still under investigation. 5% of human exons are believed to have arisen via Alu exonization events. Florea et al. have developed Alubaster, a new collection of software methods to detect such Alu exonization events in the reference genome and large-scale RNAseq data. They applied Alubaster to human frontal cortex expression data from the GTEx project and were able to identify over 800 Alu exonization events, of which around 30% were novel. Complementary to the work on the emergence of new exons, Zhang et al. have performed a comprehensive bioinformatic analysis of the evolution and expression of the U1 small nuclear ribonucleoprotein (U1 snRNP)-specific protein C (U1C) within the animal kingdom. The U1C protein is a splicing factor involved in 5’ splice-site recognition of pre-mRNA. Their analysis reveals high conservation of the gene, domain, and protein sequence and structure of U1C, which also helps to shed light on the functional impact of cancer mutations identified within the U1C gene.

Koo et al. give an example on how alternative splicing can impact cell and tissue differentiation. Koo and collegues analyzed isoform expression in the peripheral hearing organ of the chicken. With a sophisticated experimental design, they measured and compared gene and isoform expression within three different positions of the cochlea. Their analysis revealed distinct expression and splicing profiles of the splicing factors PTBP3, ESRP1, and ESRP2 along the cochlea. The importance of assessing both gene and isoform expression was also highlighted in the study of He et al., who identified isoform switches during mouse oocyte maturation. Notably, the authors found that isoform expression changes could distinguish three oocyte developmental stages more accurately than gene expression data. Furthermore, for 512 genes, the authors found significant changes in the inclusion of 3′UTR regions during oocyte maturation. Such isoform changes are important not only in homeostasis. For instance, isoform switches in cancer genes drive oncogenesis or induce resistance to cancer therapies. An example of this is illustrated by Rieger et al., who identified an ASPP2 isoform that lacks the binding site to p53, thereby promoting chemotherapy resistance and tumorigenesis in colorectal carcinomas. Thus, the overexpression of the ASPP2κ isoform could become a prognostic biomarker for some colorectal cancer treatments.

In summary, these articles highlight that much more work on alternative splicing is needed to understand its biological importance. Crucial for this is the advancement of the technologies and methods to study alternative splicing. A new promising experimental approach is long-read sequencing, which sequences full-length mRNA molecules. Thus long-read sequencing does not require the complex and error-prone, but until recently, necessary assembly step (Pollard et al., 2018). The approach is becoming widely adopted, and Paoli-Iseppi et al. provide a comprehensive review of long-read sequencing technologies, their enormous potential, and outstanding challenges. One of the significant advances highlighted by Paoli-Iseppi and colleagues is the considerable contribution of long-read technologies to the continuous improvement and completeness of the transcript annotation databases. We predict this trend will continue as long-read sequencing is increasingly applied in single-cell (Gupta et al., 2018). The increasing number of known transcripts also highlights the critical lack of functional transcript annotations. Almost no transcripts have been annotated with their biological function. Since the direct transfer of gene-level annotation to associated transcripts is not appropriate, this scarcity of transcript-level annotations presents a significant bottleneck for transcript-level analysis. Another challenge is the lack of clinical translation. While dysregulation of alternative splicing frequently plays a central role in the development of both common and rare diseases (Bonnal et al., 2020; Cummings et al., 2020), analysis of alternative splicing has yet to be widely adopted in clinical settings. Given the importance and prevalence of alternative splicing, we expect this will change within the foreseeable future.
